# 

**Published:** 1895-02

**Authors:** 


Whole Number, CCCXCIX.

New Series.

Vol. XXXIV. No. 7.

### February, 1895.

LIBRARY
Indexed
S. G. O.

### Buffalo Medical and Surgical Journal.

Established 1845 by AUSTIN FLINT, M. D.

**Editors:**

THOS. LOTHROP, M. D. WM. WARREN POTTER, M. D.

**Associate Editors:**

WILLIAM C. KRAUSS, M. D. JAMES WRIGHT PUTNAM, M. D.
JOHN A. MILLER, Ph. D. JOHN PARMENTER, M. D.

SCRIPTA FERUNT ANNOS

European Agency,
TRÜBNER & CO.
57 and 59 Ludgate Hill, London, E. C.

BUFFALO:
1895.

Foreign Subscription, $2.50

Entered at the Post-Office at Buffalo, N. Y., as Second-class Mail Matter.

*Times Printing House, Buffalo, N. Y.*

SUBSCRIPTION PRICE, $2.00 IF PAID IN ADVANCE; OTHERWISE $2.50 A YEAR.

PUBLISHED MONTHLY.

Table of Contents on Page V.

